# Supplementary material for: Major Adverse Cardiovascular Events: The Importance of Serum Levels and Haplotypes of the Anti-Inflammatory Cytokine Interleukin 10
Source: Biomolecules. 2024 Aug 9;14(8):979. doi: 10.3390/biom14080979 (PMC11353162; doi:10.3390/biom14080979)
Supplement: Supplementary file 1 [file biomolecules-14-00979-s001.zip › biomolecules-3075679-supplementary.pdf]

Table S1. Baseline characteristics of cardiovascular patients below and above detection limit of IL-10 serum level. Skewed variables were evaluated by Mann-Whitney U Test and presented as median (IQR: 25th/75th-interquartiles). Categorical variables are presented in percentage and compared by Chi-square test. (IQR: interquartile range; TIA: transient ischemic attack; MI: myocardial infarction; HDL: high density lipoprotein; LDL: low density lipoprotein).

* Yates correction.

Characteristics	Patients with IL-10 level below detection limit (n=455)	Patients with IL-10 serum level above detection limit (n=421)	p-value
Demographical and anamnestic parameters			
Age, years (median; 25/75 IQR)	68.7 (59.3/74.5)	68.2 (59.5/75.0)	0.542
Female gender (%)	23.5	28.1	0.139*
Current smoking (%)	11.6	12.8	0.668*
Body mass index, kg/m2 (median; 25/75 IQR)	28.3 (25.1/30.9)	28.1 (25.4/30.7)	0.743
History of			
Diabetes mellitus (%)	35.2	32.5	0.454*
Hypertension (%)	87.9	88.6	0.834*
MI (%)	35.2	41.1	0.083*
Stroke/TIA (%)	12.5	14.0	0.583*
Peripheral artery disease (%)	8.1	10.5	0.286*
Biochemical parameters (median; 25/75 IQR)			
C-reactive protein (mg/l),	7.3 (2.3/29.5)	10.8 (5.0/38.1)	0.001
Leukocytes (Gpt/l),	7.9 (6.4/9.7)	8.0 (6.4/9.6)	0.776
Interleukin 6 (pg/ml),	7.1 (3.5/14.1)	7.8 (3.8/17.1)	0.192
Uric acid (µmol/l)	5.7 (4.2/7.4)	5.7 (4.5/8.0)	0.128
Creatinine (mmol/l),	85 (72/106)	88 (73/109)	0.173
Total cholesterol (mmol/l),	4.4 (3.8/5.4)	4.3 (3.7/5.3)	0.150
HDL cholesterol (mmol/l),	1.0 (0.8/1.3)	1.0 (0.8/1.2)	0.113
LDL cholesterol (mmol/l),	2.7 (2.1/3.5)	2.6 (1.0/1.9)	0.137
Triglycerides (mmol/l),	1.4 (0.9/2.0)	1.4 (1.0/1.9)	0.618
